# Influence of a *5-bp Indel* Polymorphism at Promoter of the GAS5 lncRNA and Risk of Breast Cancer

**DOI:** 10.31557/APJCP.2020.21.12.3705

**Published:** 2020-12

**Authors:** Rafat Sharifi, S Shirin Shahangian, Zivar Salehi, Farhad Mashayekhi, Soheila Talesh Sasani, Laleh Mirzanezhad

**Affiliations:** *Department of Biology, Faculty of Sciences, University of Guilan, Rasht, Iran. *

**Keywords:** GAS5, lncRNA, gene polymorphism, breast cancer, genetic variation

## Abstract

Long non-coding RNAs (lncRNAs) are RNA molecules (>200 nucleotides in length) with no protein-coding capacity. Recent studies have demonstrated that lncRNAs involve in the regulation of their target genes at transcriptional, post-transcriptional and epigenetic levels. The aim of this case-control study was to explore whether growth arrest-specific *5 (GAS5) lncRNA 5-bp Ins/Del *(rs145204276) polymorphism is involved in the breast cancer susceptibility. A total of 170 cases and 220 age matched controls were recruited in this study. GAS5 lncRNA polymorphism was genotyped using tetra primers amplification refractory mutation system polymerase chain reaction (T-ARMS-PCR) method. Statistical analysis was performed using SPSS. The distribution of the genotype ins/ins, ins/del and del/del were %75.29, 21.76% and 2.94% and 52.27%, 39.55% and 8.81% in the cases and controls, respectively. The ins/del or del/del genotype had a significantly decreased risk of breast cancer as compared with the ins/ins genotype under a codominant model (OR=0.38, 95%CI 0.24-0.60, p=0.0001; OR= 0.25, 95%CI 0.09-0.69, p=0.008, respectively). Moreover, the deletion allele of this polymorphic site is associated with a protective effect (OR=0.41, 95%CI 0.28-0.60, p=0.0001). Our study provided the first evidence that the deletion allele of GAS5 rs145204276 may have a protective role in mediating individual susceptibility to breast cancer. However, further comprehensive studies are warranted in a larger sample.

## Introduction

Breast cancer ranks the first most common type of malignancies among females worldwide (Behjati et al., 2005; Torre et al., 2015). On a global scale, breast cancer accounts for approximately 450,000 dead worldwide every year (Coughlin and Ekwueme, 2009). In Iran, breast cancer accounts for 76% of all common female cancers, with an estimated prevalence rate of 23.65 per 100,000 (Harirchi et al., 2011). It has been shown that the age of breast cancer diagnosis in Iranian women is between 40 and 50 years (Kolahdoozan et al., 2010). The etiology of breast cancer comprises interactions of genetic factors and multiple environmental components. In the last years, many variants of different genes have been associated with breast cancer risk worldwide; including *p53* (Murphy et al., 2017), *IL1* (Zuo et al., 2018), *SEEP1* (Mohammaddoust et al., 2018). Numerous risk factors, includ¬ing lower age of menarche, late age of first pregnancy, fewer pregnancies, shorter or no periods of breastfeeding, later menopause, obesity, alcohol consumption, inactivity, and hormone replacement therapy (HRT), have been reported to contribute to the pathogenesis of breast cancer (Colditz and Bohlke, 2014). Yet, the underlying etiological mechanisms of breast cancer are not fully understood.

Gene regulation plays an important role in cancer development. A large portion of the human genome would transcript into noncoding RNAs (ncRNAs) which can be categorized into two major groups: house-keeping ncRNAs (such as tRNA, rRNA,) and regulatory ncRNAs (miRNA, lncRNA, etc.) (Kim and Sung, 2012). Long non-coding RNAs (lncRNAs) are RNA molecules (>200 nucleotides in length) that usually with no protein-coding capacity (Banfai et al., 2012; Iyer et al., 2015). Recent studies have demonstrated that lncRNAs involve in the regulation of their target genes at transcriptional, post-transcriptional and epigenetic levels (Schmitz et al., 2016). lncRNAs participate in diverse biological processes, such as apoptosis, cell cycle, differentiation, migration, and cell proliferation; supporting the hypothesis that dysregulation of lncRNAs may be associated with human diseases (Fang and Fullwood, 2016; Schmitz et al., 2016). In addition, studies have also revealed that some lncRNAs may function as oncogenes or tumor suppressors (Gupta et al., 2010; Fang and Fullwood, 2016). 

GAS5 LncRNA is a transcript of the growth arrest-specific 5 (*GAS5*) gene, which was first identified in 1988 (Pickard and Williams, 2015). The *GAS5* gene is located on chromosome 1q25.1 that encodes several small nucleolar RNAs (snoRNAs), microRNAs (miRNAs), PIWI-interacting RNAs (piRNAs) and lncRNA (Smith and Steitz, 1998; Brameier et al., 2011; He et al., 2015).

Downregulation of GAS5 has been reported in several malignancies, including breast, gastric and prostate cancer (Mourtada-Maarabouni et al., 2009; Pickard et al., 2013; Sun et al., 2014); thus, it may function as a tumor suppressor (Yu and Li, 2015). Although the exact mechanism of GAS5 lncRNA is not completely understood, it is suggested that it repress transcriptional activity by competing with DNA glucocorticoid receptor response element (GRE) for binding to the glucocorticoid receptors (GR) (Kino et al., 2010). In addition, *GAS5 *can regulate target genes by direct binding. It has been shown that GAS5 can negatively regulate translation of the c-MYC mRNA and other transcription factors (Hu et al., 2014). It should be noted that other mechanisms such as direct interaction with miR-21, CDK6, YBX1 and other proteins are also important (Pickard and Williams, 2015). 

A genetic polymorphism is a variation in DNA sequence that occurs in a population at a frequency greater than 1%, resulting in the existence of multiple alleles. The most famous polymorphic site of GAS5 is rs145204276, which contains a 5-base pair insertion/deletion (indel) polymorphism in the promoter region with a minor allele frequency (MAF) 0.12 (Tao et al., 2015). The indel polymorphisms likely have a great influence on human property and disease susceptibility (Mullaney et al., 2010). It has been shown that the relative GAS5 expression level in del/del genotype was significantly higher than ins/del and ins/ins genotype (Tao et al., 2015). In recent years, the *GAS5 lncRNA* gene polymorphisms have been reported to be an important predisposition factor to gastric cancer (Li et al., 2018), acute myeloid leukemia (Yan et al., 2017) and hepatocellular carcinoma (Tao et al., 2015). However, the relationship between GAS5 lncRNA indel variation and the risk of breast cancer has not been investigated to date. Thus, the aim of this case-control study was to investigate the asso¬ciation between a 5-base pair indel polymorphism (rs145204276) of GAS5 lncRNA and breast cancer risk in Iranian population.

## Materials and Methods

*Subjects*

We conducted a case-control study on 390 women (170 cases and 220 controls) who were recruited between August 2013 and December 2017. Breast cancer patients diagnosed either by imaging evidence, histopathologic documentation, with a minimum age of 35 years. Pathobiological features (histology type, lymph node involvement and hormone receptor status) were obtained from the medical records of the hospitals and collected. Patients who had previous radiotherapy or chemotherapy were excluded in the study. In addition, 220 age-matched healthy women who got a routine health checkup and normal mammography without any history of neoplasms or chronic disease were recruited as controls. Characteristics of all enrolled subjects, such as age, breast feeding history, menopausal status and using oral contraceptive pill (OCP) were collected. Written informed consent for the genetic analysis was obtained from each subject participating in the study. The study was conducted in accordance with the Declaration of Helsinki regarding the use of human samples.

*Genotyping*

Blood samples of breast cancer cases and controls were collected in EDTA-containing tubes and centrifuged at 1500 g for 10 minutes. Plasma and buffy coat were separated and stored at -20°C. Genomic DNA was extracted using GPP Solution Kit (Gene Pajoohan, Iran) according to the manufacturer’s recommendations. Purity and quantity of DNA were checked with a spectrophotometer (Thermo Scientific, USA) with A260/280 1.75–1.85. All DNA samples were dissolved in TE buffer (5 mM Tris–HCl, 0.1 mM EDTA, pH 8.5) with a final concentration ranging from 55–365 ng/µl and stored at -20°C until use. 

The GAS5 lncRNA polymorphism was genotyped using tetra primers amplification refractory mutation system polymerase chain reaction (T-ARMS-PCR) method. Primers were designed by Oligo7 software (7.60 version, Molecular Biology Insights Inc., Cascade, CO, USA). Primers were synthesized by MWG-Biotech (Ebersberg, Germany). The primers sequences were as follows: Ins-F (5ʹ- GCAGAGACATGACCGTCCAC- 3ʹ) and ins-R (5ʹ-CCCCATCCCCAGAGCTTTCGTT-3ʹ) for ins allele; Del-F (5ʹ-AAAACCCGCAACATTCGCAAA-3ʹ) and del-R (5ʹ-CCCCATCCCCAGAGCTTTCGTC-3ʹ) for del allele. PCR was carried out on the thermal cycler (BioRad, USA) in a total volume of 20 µl, including 50 ng genomic DNA, 1X PCR buffer, 3 mM MgCl_2_, 2 mM of each dNTP, 0.5 mM/ml primer mix, and 1 U Taq DNA polymerase (Gene Fanavaran, Iran). The cycling profile was as follows: initial denaturation at 95°C for 6 minutes, 35 cycles of denaturation at 94°C for 35 seconds, annealing at 56°C (ins allele) and 60°C (del allele) for 40 seconds, with a final extension step at 72°C for 2 minutes. The fragment lengths were 109 and 348 bp for ins and del alleles, respectively. The amplicons were electrophoresed with a 50 bp DNA ladder (SMOBIO Technology, Inc, Taiwan) on 1.5% agarose gel containing safe stain. All blood samples were genotyped successfully. Genotyping was performed in a blinded manner.

*Statistical analysis*

The OpenEpi software (www.OpenEpi.com) based on minor allele frequency (MAF) of SNP was used to calculate a required sample size. Calculations of Hardy-Weinberg equilibrium (HWE) were conducted using the chi-square test to compare the observed genotype frequencies with the expected frequencies among the case and control subjects. HWE graphically analyzed by using triangular ternary plot through “de Finetti” diagram (http://ihg.gsf.de/cgi-bin/hw/hwa1.pl). Allele frequencies of the *GAS5 lncRNA* polymorphisms were estimated by gene counting. Within the cancer cases and controls, frequencies of GAS5 lncRNA different alleles and genotypes investigated were calculated using the chi-square test. Odds ratios (OR) were calculated to study the association between alleles or genotypes of GAS5 lncRNA and risk of breast cancer by the means of the Fisher’s exact test with the reference category being the homozygote of the major allele among the subjects. We also tested four different genetic models including codominant, dominant, recessive and overdominant. Statistical analyses were performed using SPSS version 16.0 for Windows (SPSS, Inc., Chicago, IL, USA). Significance was set at p<0.05. 

## Results

The demographic characters of the subjects are presented in Table 1. The breast cancer patient’s ages ranged between 36 and 61 years. The age range in the controls was from 30 to 63 years. No significant difference was detected for age group between breast cancer cases and healthy controls (P>0.05). As shown in Table 1, we found significant differences in the breastfeeding history (P = 0.001) and using oral contraceptive (P = 0.001) when the groups were compared. We also observed that 68% of the patients displayed ductal histology, and 73% of the patients exhibited stage III-IV tumors. 

**Figure 1:**
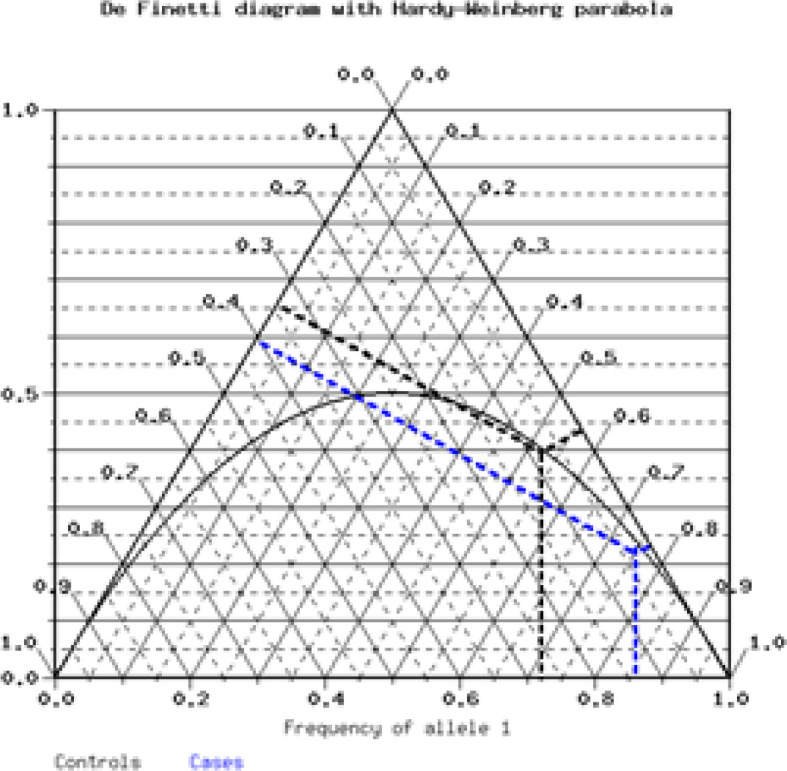
De Finetti Ternary Diagrams of GAS5 lncRNA rs145204276 Polymorphism. A ternary diagram for GAS5 lncRNA polymorphism with a Hardy-Weinberg parabola for BC cases (Blue line) and controls (black line)

Genotyping for rs145204276 of GAS5 lncRNA was performed in 170 breast cancer patients and 220 healthy controls. The Hardy–Weinberg analysis is shown in Table 2 and Figure 1 where the distribution of genotypic frequencies is described. The observed genotypes distribution was in HWE suggesting these subjects could represent the total population. A significant difference was found between genotype frequencies of the GAS5 polymorphism in the control and the patient groups determined by the expected value of the *χ*^2^-test (P=0.0001). The distribution of the genotype ins/ins, ins/del and del/del were %75.29, 21.76% and 2.94% and 52.27%, 39.55% and 8.81% in the cases and controls, respectively. As shown in Table 3, the ins/del or del/del genotype had a significantly decreased risk of breast cancer as compared with the ins/ins genotype under a codominant model (OR=0.38, 95%CI 0.24-0.60, p=0.0001; OR= 0.25, 95%CI 0.09-0.69, p=0.008, respectively). Similar results were also observed in other genetic models. Moreover, the deletion allele of this polymorphic site is associated with a protective effect (OR=0.41, 95%CI 0.28-0.60, p=0.0001).

The association between clinic-pathological char¬acteristics of breast cancer patients with GAS5 polymorphism is shown in Table 4. The ins/ins genotype was more frequent in patients with larger tumor size, ductal adenocarcinoma, higher stage, lymph node metastasis as well as ER and PR positive status. Patients with ins/del genotype had a lower risk of breast cancer developing with tumor size >5 cm, lymph node involvement and higher stage of disease. We did not observe any association between tumor site, ER, PR and Her2 status and distribution of GAS5 lncRNA genotypes (p>0.05). 

**Table 1:** Baseline Characteristics of Case-Control Study Population

Characteristic	Case N(%)	Control N(%)	P-value
Age (Mean±SD*)	49.1± 12.8	49.7± 14.3	0.63
Breast-feeding history			
Positive	127 (74.70)	188 (85.45)	0.001
Negative	43 (25.30)	32 (14.54)	
Menopausal status			
Premenopausal	66 (38.82)	87 (39.55)	0.78
Postmenopausal	104 (61.18)	133 (60.45)	
Using oral contraceptive			
Yes	60 (35.29)	52 (23.64)	0.001
No	110 (64.71)	168 (76.36)	
Estrogen receptor			
Positive	109 (64.11)	-	-
Negative	61 (35.89)	-	-
Progesterone receptor			
Positive	119 (70)	-	-
Negative	51 (30)		
Her2 receptor			
Positive	48 (28.23)	-	-
Negative	122 (71.77)		
Tumor size			
5cm>	117 (68.82)	-	-
5cm<	53 (31.18)	-	-
Metastasis to lymph node			
Yes	120 (70.59)	-	-
No	50 (29.41)	-	-

*, Standard deviation

**Table 2:** Description of HWE for BC Cases and Controls

Polymorphic site	Cases N=170	Controls N=220
rs145204276	Ins/Ins=128	Ins/Ins=115
	Ins/Del=37	Ins/Del=87
	Del/Del=5	Del/Del=18
	ƒ(a1)=0.86±0.020	ƒ(a1)=0.72±0.022
	F=0.08649	F=0.01823
	p (Pearson)=0.259472	p (Pearson)=0.786800
	p (Llr)=0.284453	p (Llr)=0.787389
	p (Exact)=0.325168	p (Exact)=0.740879

F, Inbreeding coefficient; p(Pearson), Pearson’s goodness-of-fit chi-square (degree of freedom=1); p(Llr), Log likelihood ratio chi-square (degree of freedom=1); p(Exact), Exact test

**Table 3:** The Allele and Genotype Frequencies of the GAS5 lncRNA (rs145204276) and BC Risk

Genetic Model	Genotypes	Patients N (%)	Controls N (%)	OR* (95%CI**)	P-value
Codominant	Ins/ Ins	128 (75.29)	115 (52.27)	1.00(Ref)	-
	Ins/ Del	37 (21.76)	87 (39.55)	0.38 (0.24-0.60)	0.0001
	Del/ Del	5 (2.94)	18 (8.81)	0.25 (0.09-0.69)	0.008
Dominant	Ins/ Ins	128 (75.29)	115 (52.27)	1.00 (Ref)	-
	Ins/ Del + Del/ Del	42 (24.71)	105 (47.73)	0.36 (0.23-0.56)	0.001
Recessive	Ins/ Ins + Ins/ Del	165 (97.06)	202 (91.82)	1.00 (Ref)	-
	Del/ Del	5 (2.94)	18 (8.18)	0.34 (0.12-0.93)	0.037
Overdominant	Del/ Del+Ins/ Ins	133 (78.24)	133 (60.45)	1.00(Ref)	-
	Ins/ Del	37 (21.76)	87 (39.55)	0.42 (0.27-0.67)	0.0002
Alleles	Ins	293 (0.86)	317 (0.72)	1.00(Ref)	-
	Del	47 (0.14)	123 (0.28)	0.41 (0.28-0.60)	0.0001

*OR, odds ratio; **CI, confidence interval.

**Table 4:** Association of GAS5 lncRNA Polymorphism with Breast Cancer Clinical Characteristics

Variables	Ins/Ins	Ins/Del	Del/Del
	n=128	n=37	n=5
Tumor size (Cm)			
≥5/<5	95/33	20/17	2/3
OR(95%CI)	Ref	0.40 (0.19-0.87)	0.23 (0.03-1.44)
P-value		0.02	0.11
Tumor site			
Ductal/Lobular	91/37	22/15	3/2
OR(95%CI)	Ref	0.59 (0.28-1.27)	0.61 (0.09-3.80)
P-value		0.18	0.59
Stage			
III-IV/0-II	101/27	22/15	2/3
OR(95%CI)	Ref	0.39 (0.17-0.85)	0.17 (0.02-1.12)
P-value		0.01	0.06
Nodal status			
Positive/Negative	98/30	21/16	1/4
OR(95%CI)	Ref	0.40 (0.18-0.86)	0.07 (0.01-0.71)
P-value		0.02	0.02
Estrogen receptor			
Positive/Negative	83/45	23/14	3/2
OR(95%CI)	Ref	0.89 (0.41-1.89)	0.81 (0.13-5.04)
P-value		0.76	0.82
Progesterone receptor			
Positive/Negative	91/37	25/12	3/2
OR(95%CI)	Ref	084 (0.38-1.86)	0.60 (0.09-3.80)
P-value		0.67	0.59
HER2 status			
Positive/Negative	35/93	26-Nov	2/3
OR(95%CI)	Ref	1.12 (0.50-2.51)	1.77 (0.28-11.05)
P-value		0.77	0.54

## Discussion

In this case-control study, we studied the impact of GAS5 lncRNA (rs145204276) polymorphism in breast cancer susceptibility in Iranian population. To our knowledge, this is the first study in Iranian population showing a significant association between the GAS5 lncRNA (rs145204276) del allele and decreased breast cancer susceptibility. Moreover, the ins/ins homozygous breast cancer patients presented significantly more often with Ductal carcinoma than patients carrying a del allele of GAS5. 

There are no previous reports about the prevalence of the rs145204276 variant in patients with breast cancer. A previous study conducted by Zheng et al. demonstrated that the deletion allele of rs145204276 was significantly associated with 21% decreased risk of colorectal adenocarcinoma and compared with the genotype ins/ins, both the genotype ins/del and del/del showed decreased susceptibility (Zheng et al., 2016). However, a matched hospital-based case–control study with 813 colorectal cancer and 926 cancer-free controls supported the hypothesis that del allele of GAS5 indel polymorphism played a role in the pathogenesis of CRC in the Chinese population (Zhu et al., 2016). A recently published data from Xu and colleagues showed that del/del genotype had a significantly higher expression of GAS5 in patients with osteosarcoma (Xu et al., 2018). In addition, cases with del/del genotype were found to have evidently hypermethylation at the 7^th^ CpG site as compared with those with genotype ins/ins.

Increasing evidence has suggested that lncRNAs are key players in various processes of breast cancer, such as proliferation, metastasis, angiogenesis and drug resistance (Wang et al., 2017). In addition to downregulation of GAS5 lncRNA in some tumors, suppression of GAS5 expression has been found to correlate with tumor size and advanced disease in lung (Tan et al., 2017), gastric (Guo et al., 2015), colon (Li et al., 2017b) and ovarian (Li et al., 2016) cancers. GAS5 transcripts induce growth arrest and cell death in human breast cancer cell lines, suggesting that GAS5 lncRNA may have a tumor-suppressive role in breast cancer cells (Mourtada-Maarabouni et al., 2009). However, GAS5 may act as a proto-oncogene in HCC (Tao et al., 2015). 

The GAS5 rs145204276 is a 5-bp indel polymorphism (-/AGGCA) in the gene promoter region. It has been shown that the deletion allele of rs145204276 could alter the methylation status of CpG islands which could increase the expression of lncRNA in lung cancer and hepatocellular carcinoma (Tao et al., 2015; Li et al., 2017a). Previous study showed that rs145204276 may contribute to hepatocarcinogenesis by affecting methylation status of the GAS5 promoter and subsequently its transcriptional activity. 

To date, there have been only two previous studies to establish an association between lncRNA genetic variants and breast cancer susceptibility in Iranian women. Hassanzarei and coworkers reported that HOX transcript antisense intergenic RNA (HOTAIR) rs920778, rs12826786, and rs1899663 polymorphisms may be associated with breast cancer risk in a sample of southeast Iranian population (Hassanzarei et al., 2017). In a case-control study involving 122 breast cancer cases and 200 normal age-match female, Khorshidi et al, suggested that breast cancer risk is significantly associated with ANRIL variants (Khorshidi et al., 2017).

Some limitations of this study should be noted. Firstly, this study only considers a north Iranian population that may limit the application of these findings to other populations. Second, the sample size was small; the results should be interpreted with caution. Given the diversity of rs145204276 polymorphism, a larger number of subjects are required to evaluate the role of GAS5 lncRNA polymor¬phism in breast cancer risk. Third, other polymorphisms of GAS5 and their possible interactions were not evaluated. However, numerous factors act individually and together to influence the risk of breast cancer. So, we should involve more factors in our future work.

In conclusion, our study provided the first evidence that the del allele of GAS5 rs145204276 may have a protective role in mediating individual susceptibility to breast cancer. As breast cancer is a complex disease that involves environmental and genetic factors, further comprehensive studies are warranted in a larger sample, including investigation of combined effects of multiple loci other than one polymorphic site or single gene.
